# Retraction: Effects of Eicosapentaenoic Acid and Docosahexaenoic Acid on Prostate Cancer Cell Migration and Invasion Induced by Tumor-Associated Macrophages

**DOI:** 10.1371/journal.pone.0173325

**Published:** 2017-02-28

**Authors:** Cheng-Chung Li, Yu-Chen Hou, Chiu-Li Yeh, Sung-Ling Yeh

A reader raised concerns about potential data duplication in several figures. Specifically, the reader identified regions of overlap among the following figure panels:

- Panels E25, E50, and D50 shown in Figure 4
- Panels C and D25 panels in Figure 4
- Panel C in Figure 4 (Migration) and Panel C in Figure 5 (Invasion)

The authors acknowledged that there was an error in the preparation of these figures. However, they no longer have the data underlying the results shown in the panels in Figure 4, and they were unable to replicate these results using the same experimental conditions.

Due to the concerns about the integrity of the results, and the absence of data reproducing the findings, the authors have concerns about the validity and robustness of the results reported and have requested the retraction of this article.

The authors apologize to the readers and editors of *PLOS ONE*.
